# Arsenic-induced dyslipidemia in male albino rats: comparison between trivalent and pentavalent inorganic arsenic in drinking water

**DOI:** 10.1186/s40360-015-0015-z

**Published:** 2015-06-05

**Authors:** Olusegun K. Afolabi, Adedoja D. Wusu, Olabisi O. Ogunrinola, Esther O. Abam, David O. Babayemi, Oluwatosin. A. Dosumu, Okechukwu. B. Onunkwor, Elizabeth. A. Balogun, Olusegun O. Odukoya, Oladipo Ademuyiwa

**Affiliations:** Department of Biochemistry, Federal University of Agriculture, Abeokuta, Nigeria; Department of Chemistry, Federal University of Agriculture, Abeokuta, Nigeria; Department of Biochemistry, Ladoke Akintola University of Technology, Ogbomosho, Nigeria; Department of Biochemistry, Lagos State University, Ojoo, Lagos, Nigeria; Department of Biochemistry, University of Ilorin, Ilorin, Nigeria; Biochemistry Unit, Department of Chemical Sciences, Bells University of Technology, Ota, Nigeria

**Keywords:** Trivalent inorganic arsenic, Pentavalent inorganic arsenic, Drinking water, Dyslipidemia

## Abstract

**Background:**

Recent epidemiological evidences indicate close association between inorganic arsenic exposure via drinking water and cardiovascular diseases. However, the exact mechanism of this arsenic-mediated increase in cardiovascular risk factors remains enigmatic.

**Methods:**

In order to investigate the effects of inorganic arsenic exposure on lipid metabolism, male albino rats were exposed to 50, 100 and 150 ppm arsenic as sodium arsenite and 100, 150 and 200 ppm arsenic as sodium arsenate respectively in their drinking water for 12 weeks.

**Results:**

Dyslipidemia induced by the two arsenicals exhibited different patterns. Hypocholesterolemia characterised the effect of arsenite at all the doses, but arsenate induced hypercholesterolemia at the 150 ppm As dose. Hypertriglyceridemia was the hallmark of arsenate effect whereas plasma free fatty acids (FFAs) was increased by the two arsenicals. Reverse cholesterol transport was inhibited by the two arsenicals as evidenced by decreased HDL cholesterol concentrations whereas hepatic cholesterol was increased by arsenite (100 ppm As), but decreased by arsenite (150 ppm As) and arsenate (100 ppm As) respectively. Brain cholesterol and triglyceride were decreased by the two arsenicals; arsenate decreased the renal content of cholesterol, but increased renal content of triglyceride. Arsenite, on the other hand, increased the renal contents of the two lipids. The two arsenicals induced phospholipidosis in the spleen. Arsenite (150 ppm As) and arsenate (100 ppm As) inhibited hepatic HMG CoA reductase. At other doses of the two arsenicals, hepatic activity of the enzyme was up-regulated. The two arsenicals however up-regulated the activity of the brain enzyme. We observed positive associations between tissue arsenic levels and plasma FFA and negative associations between tissue arsenic levels and HDL cholesterol.

**Conclusion:**

Our findings indicate that even though sub-chronic exposure to arsenite and arsenate through drinking water produced different patterns of dyslipidemia, our study identified two common denominators of dyslipidemia namely: inhibition of reverse cholesterol transport and increase in plasma FFA. These two denominators (in addition to other individual perturbations of lipid metabolism induced by each arsenical), suggest that in contrast to strengthening a dose-dependent effect phenomenon, the two forms of inorganic arsenic induced lipotoxic and non-lipotoxic dyslipidemia at “low” or “medium” doses and these might be responsible for the cardiovascular and other disease endpoints of inorganic arsenic exposure through drinking water.

## Background

Among the plethora of toxicants, arsenic ranks as an environmentally ubiquitous and epidemiologically important metalloid currently poisoning tens of millions of people worldwide [1, 2]. It is found in both inorganic and organic forms and in different valence or oxidation states in the environment.

Chronic exposure to arsenic is associated with a wide range of toxic effects [1, 3]. Cancer of the skin, lung, kidney, liver and urinary bladder are the important cancers associated with these toxic effects [1–3]. Among the non-cancer effects of arsenic, diabetes mellitus, goitre, hepatomegaly, bronchitis, bronchiectasis, cerebrovascular and cardiovascular diseases, are well documented [3–5]. Epidemiological studies have demonstrated that ingestion of arsenic through drinking water might be responsible for these carcinogenic and non-cancer effects of arsenic [3, 4, 6]. In spite of this large body of information about the toxic effects of arsenic, the precise mechanisms of action for the many disease endpoints following acute and chronic arsenic exposure, as well as the threshold for biologic effects and disease risks, remain enigmatic [1].

Over the years, epidemiological studies have identified lipid and lipoprotein abnormalities as independent risk factors in the pathogenesis and progression of atherosclerosis and cardiovascular diseases [7, 8]. There is also increasing evidence that environmental factors/contaminants (most especially heavy metals) contribute to this dyslipidemia [7, 9, 10]. Studies conducted in arsenic-exposed populations revealed a prevalence of nearly a full spectrum of cardiovascular diseases including hypertension, atherosclerosis, blackfoot disease, ischaemic heart diseases, etc. [3, 4]. Therefore, it seems reasonable to hypothesise that the association between arsenicosis and cardiovascular diseases may be mediated through modification of lipid and lipoprotein metabolism. The work reported here explored this hypothesis.

## Materials and methods

### Chemicals

Sodium arsenite and sodium arsenate were products of Sigma-Aldrich, Missouri, USA.

### Animals and treatment

Experimental protocols were conducted in accord with guidelines of the Institutional Animal Care and Use Committee and were approved by the Animal Ethical Committee of the Department of Biochemistry, Federal University of Agriculture, Abeokuta, Nigeria.

Fifty-six male Wistar rats (bred in the College of Veterinary Medicine, Federal University of Agriculture, Abeokuta, Nigeria) with a mean body weight of 130 g were used for the experiments. They were housed in an animal room with normal controlled temperature (22 ± 2 °C) and a regular 12 h light–dark cycle (06:00–18:00 h). They were allowed 14 days to acclimatise before the commencement of arsenic exposure. The animals were maintained on a standard pellet diet.

Animals were divided into 8 groups of 7 animals each. While 2 groups served as control and received distilled water, the remaining groups (3 groups each) were exposed to 50, 100 and 150 ppm arsenic as sodium arsenite and 100, 150 and 200 ppm arsenic as sodium arsenate respectively in their drinking water for 12 weeks. These arsenic concentrations were chosen based on previous studies [11–15]. At the end of arsenic exposure, blood was collected from the animals into heparinised tubes by cardiac puncture under light ether anaesthesia after an overnight fast. Liver, kidney, heart, lung, brain and spleen were also removed from the animals for arsenic and lipid analyses. An aliquot of the blood samples was taken for arsenic determination while the remaining blood samples were centrifuged to separate plasma and red blood cells. All samples were stored at −20 °C until analysed.

### Arsenic determination

A portion of the frozen organs (≈200 mg) and whole blood (0.2 ml) were digested in nitric acid. Total arsenic (which would include inorganic and organic forms) was determined using atomic absorption spectrometry. Results are expressed as μg As/ml for blood and μg As/g wet weight for the organs.

### Biochemical analyses

#### Plasma and lipoprotein lipid profiles

Determination of the major lipids (cholesterol, triglycerides, phospholipids and free fatty acids) in plasma and lipoproteins followed established procedures. Details of these have been given in our earlier studies [7, 16–19].

#### Organ and erythrocyte lipid profiles

Lipids were extracted from the organs (liver, kidney, heart, lung, brain and spleen) as described by Folch et al. (1957) [20] while extraction of erythrocyte lipids followed the procedure described by Rose and Oklander (1965) [21]. After washing with 0.05 M KCl solution, aliquots of the lipid extracts were then used for the determination of lipid profiles. Details of these are given as reported earlier [7, 16–19].

#### Determination of hepatic and brain HMG-CoA reductase activity

This was determined according to the method of Rao and Ramakrishnan (1975) [22] by measuring the hepatic and brain concentrations of HMG-CoA and mevalonate. The ratio of HMG-CoA to mevalonate is taken as an index of the activity of HMG-CoA reductase. An increase in this ratio indicates inhibition of cholesterogenesis while a decrease indicates enhanced cholesterogenesis.

#### Statistical analysis

Results are expressed as mean ± SEM. One way analysis of variance (ANOVA) followed by Tukey’s test was used to analyze the results with *p* < 0.05 considered significant. Associations among the parameters and their magnitudes were determined using Pearson correlation.

## Results

Table 1 depicts the index of arsenic exposure (expressed as weekly arsenic intake/rat) calculated by the formula: water consumption (ml) × arsenic concentration (ppm). Arsenic intake per week was relatively constant in each dose group. For the arsenite-exposed rats, each animal ingested 7.41 ± 0.29 mg As/week in the 50 ppm group, while arsenic ingestion was 13.13 ± 0.69 mg As/week and 17.33 ± 0.60 mg As/week in the 100 ppm and 150 ppm groups respectively. For the 12 weeks of exposure, each animal in the 50 ppm group ingested a total of 88.91 mg of arsenic, while an animal in the 100 ppm group and 150 ppm group had a total arsenic intake of 157.58 mg and 207.90 mg respectively. The arsenate-exposed animals also showed a constant rate of arsenic intake. In the 100 ppm group, an animal consumed on the average, a total of 12.06 ± 0.68 mg As/week, while in the 150 ppm and 200 ppm groups, the intake per week were 16.02 ± 1.07 mg and 22.04 ± 1.87 mg respectively. Total arsenic intake in the groups amounted to 144.75 mg, 192.20 mg and 264.42 mg per rat for the 100 ppm, 150 ppm and 200 ppm doses respectively.

**Table 1:** Weekly and total arsenic intakes of each animal on exposure to sodium arsenite and sodium arsenate for 12 weeks through drinking water

Week	Sodium arsenite			Sodium arsenate		
	Arsenic Dose			Arsenic Dose		
	50 ppm	100 ppm	150 ppm	100 ppm	150 ppm	200 ppm
1	9.30	16.00	18.30	16.31	23.27	37.03
2	8.53	16.83	21.30	16.26	21.69	29.94
3	8.57	17.34	19.29	11.80	17.57	26.75
4	8.26	13.54	16.84	12.69	18.56	23.94
5	6.89	13.60	15.43	13.26	15.63	22.06
6	6.84	12.40	16.54	12.49	15.21	19.94
7	6.94	11.77	18.47	10.06	16.67	20.09
8	6.44	10.30	15.60	11.94	13.76	17.73
9	6.49	11.08	15.21	9.86	12.94	17.08
10	6.53	10.97	14.61	9.57	11.91	18.60
11	7.50	11.09	19.59	11.38	12.30	14.67
12	6.63	12.72	16.71	9.14	12.69	16.60
Total intake (mg)	88.91	157.58	207.90	144.75	192.20	264.42
Mean intake/week (mg) (± S.E.M)	7.41 ± 0.29	13.13 ± 0.69	17.33 ± 0.60	12.06 ± 0.68	16.02 ± 1.07	22.04 ± 1.87

Arsenic levels in some of the tissues in rats administered either arsenite or arsenate in their drinking water are shown in Table 2. Although the administration of the two arsenicals resulted in an accumulation of arsenic in blood, liver, kidney, heart, lung, brain and spleen of the animals, only in the kidney of arsenate-treated and the brain of arsenite-treated animals was the accumulation found to be dose-dependent. In arsenite-treated animals, exposure to an arsenic dose of 50 ppm resulted in arsenic content of 305 μg As/ml of blood. Doubling of the arsenic dose did not result in any further increase in arsenic content. Rather, arsenic content of the blood approached a saturation. At the highest dose of arsenic in the sodium arsenite group (150 ppm), arsenic content of the blood reduced from 301 μg As/ml to 258 μg As/ml. In the arsenate-treated animals, exposure to an arsenic dose of 100 ppm resulted in an arsenic content of 228.44 μg As/ml of blood. Increasing the arsenic dose resulted in a further increase in arsenic content from 228.44 μg As/ml to 267.10 μg As/ml. Increasing the arsenic dose to 200 ppm did not result in any appreciable increase in arsenic content of blood. Rather, arsenic content of blood in this group of animals peaked at 273.23 μg As/ml. In arsenite-exposed animals, the spleen accumulated the highest amount of arsenic, whereas in arsenate-exposed animals, kidney accumulated the highest amount of arsenic.

**Table 2:** Arsenic concentrations in the tissues of the animals on exposure to sodium arsenite and sodium arsenate for 12 weeks through drinking water

	Sodium arsenite				Sodium arsenate			
	Arsenic dose				Arsenic dose			
Tissue	Control	50 ppm	100 ppm	150 ppm	Control	100 ppm	150 ppm	200 ppm
Blood (μg/ml)	11.24 ± 2.35a	304.99 ± 15.43b	300.85 ± 18.89b	257.62 ± 12.61c	23.60 ± 2.99a	228.44 ± 31.07b	267.10 ± 13.79c	273.23 ± 14.33c
Liver (μg/g wet weight)	1.73 ± 0.07a	19.92 ± 2.16b	14.85 ± 1.07c	22.44 ± 3.02b	3.54 ± 0.26a	10.03 ± 0.58b	19.32 ± 3.93c	14.19 ± 0.96d
Kidney (μg/g wet weight)	2.06 ± 0.16a	5.56 ± 0.28b	39.54 ± 1.95c	27.36 ± 2.03d	2.06 ± 0.16a	6.31 ± 0.37b	32.84 ± 3.09c	47.90 ± 2.99d
Brain (μg/g wet weight)	0.69 ± 0.05a	3.11 ± 0.24b	24.88 ± 2.57c	35.21 ± 4.33d	1.74 ± 0.15a	18.95 ± 1.81b	31.65 ± 2.15c	22.11 ± 2.10d
Heart (μg/g wet weight)	0.20 ± 0.03a	16.54 ± 1.11b	53.54 ± 5.57c	46.01 ± 2.30c	0.02 ± 0.03a	42.16 ± 2.39c	18.80 ± 3.46b	27.98 ± 2.99b
Lung (μg/g wet weight)	0.71 ± 0.03a	4.97 ± 0.45a	28.73 ± 3.73b	33.20 ± 4.67b	0.75 ± 0.02a	6.64 ± 0.88a	44.80 ± 7.18b	28.39 ± 4.62b
Spleen (μg/g wet weight)	3.37 ± 0.29a	40.35 ± 2.92b	38.08 ± 1.60b	58.32 ± 5.08c	5.88 ± 0.33a	20.66 ± 2.14b	31.41 ± 3.40b	23.12 ± 1.06b

Each value represents the mean ± S.E.M. of 7 rats. Values within a row with different alphabets for each arsenic compound are significantly different at p < 0.05

**Fig. 1:**
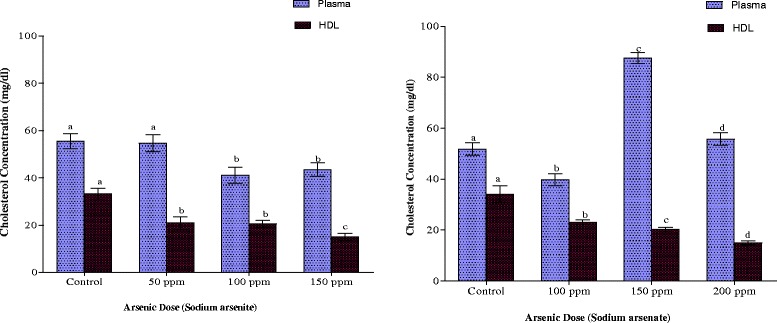
Effects of arsenite and arsenate on plasma and HDL cholesterol concen-trations. Each bar represents the mean ± S.E.M. of 7 rats. Bars with different alphabets are significantly different at p < 0.05

Dyslipidemia induced by arsenite was characterised by hypocholesterolemia at all the arsenic doses (Fig. 1). At both 100 ppm and 150 ppm doses of arsenic in arsenite-treated animals, cholesterol reduced from 56.48 ± 3.21 mg/dl in control to 41.10 ± 3.35 mg/dl and 43.49 ± 3.87 mg/dl respectively. Arsenate, on the other hand, increased plasma cholesterol by 69 % at the 150 ppm As dose but decreased this lipid by 23 % at 100 ppm As. HDL cholesterol was reduced to the same extent by the two arsenicals. At the highest dose of each arsenical, HDL cholesterol was just about 50 % of control values (Fig. 1).

**Fig 2:**
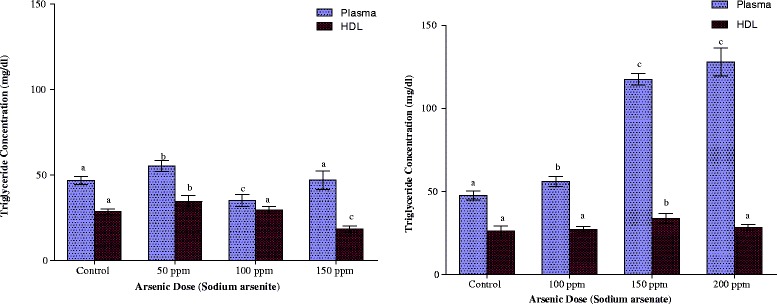
Effects of arsenite and arsenate on plasma and HDL triglyceride concen-trations. Each bar represents the mean ± S.E.M. of 7 rats. Bars with different alphabets are significantly different at p < 0.05

As indicated in Fig. 2, arsenite elicited a hormetic response in plasma triglyceride concentration in which the 50 ppm As dose up-regulated whereas the 100 ppm As dose down-regulated the plasma triglyceride content. The 50 ppm arsenic dose in arsenite-exposed animals increased the plasma triglyceride content from 46.81 ± 2.25 to 55.32 ± 3.33 mg/dl. Doubling the arsenic dose resulted in a 25 % reduction in triglyceride whereas plasma triglyceride level at highest arsenic dose was not significantly different from the control (p > 0.05). Arsenate, on the other hand, induced hypertriglyceridemia at all the doses of arsenic employed. Arsenite exposure at the 50 ppm As dose also caused an elevation of the HDL triglyceride content while, at 150 ppm As dose, the lipid was significantly reduced (p < 0.05). With arsenate exposure, however, there was no significant change in HDL triglyceride concentrations except at the 150 ppm As dose where an increase in concentration was observed.

**Fig. 3:**
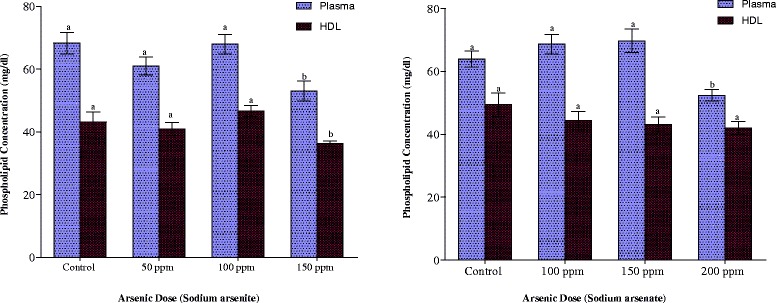
Effects of arsenite and arsenate on plasma and HDL phospholipid concentrations. Each bar represents the mean ± S.E.M. of 7 rats. Bars with different alphabets are significantly different at p < 0.05

Plasma phospholipid concentrations were significantly reduced in both arsenite- and arsenate-exposed rats at the highest dose (p < 0.05) while no significant changes at both the low and medium doses were observed (Fig. 3). Exposure of the animals to either arsenite or arsenate did not result in any significant changes in the HDL phospholipid contents at all the doses employed, although there was a gradual depression of the concentrations as the arsenate doses were increased (Fig. 3).

**Fig. 4:**
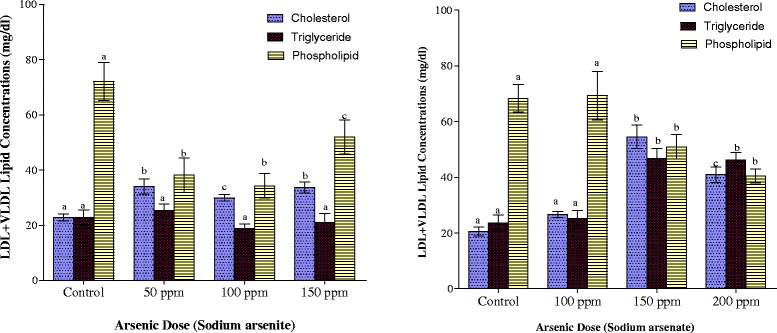
Effects of arsenite and arsenate on LDL + VLDL cholesterol, triglyceride and phospholipid concentrations. Each bar represents the mean ± S.E.M. of 7 rats. Bars with different alphabets are significantly different at p < 0.05

Cholesterol concentration in the LDL + VLDL fraction was increased on exposure to arsenite and arsenate at all arsenic doses (Fig. 4). The increase in the arsenite-exposed rats amounted to 49 % whereas in arsenate-exposed rats the increases amounted to 30 %, 165 % and 99 % in the 100 ppm, 150 ppm and 200 ppm groups respectively. Triglyceride contents of the LDL + VLDL fraction were not affected by exposure to arsenite but arsenate caused a 2-fold increase in same lipid at 150 ppm and 200 ppm As doses (Fig. 4).

The mean phospholipid concentrations in LDL + VLDL as depicted in Fig. 4 indicate that both arsenite and arsenate exposures resulted in depletion of LDL + VLDL phospholipids. In arsenite-exposed animals, the highest depletion of 52 % was observed at the 100 ppm As dose, but with arsenate, the reduction was maximal in the highest dose group (200 ppm) where 41 % reduction was observed.

**Fig. 5:**
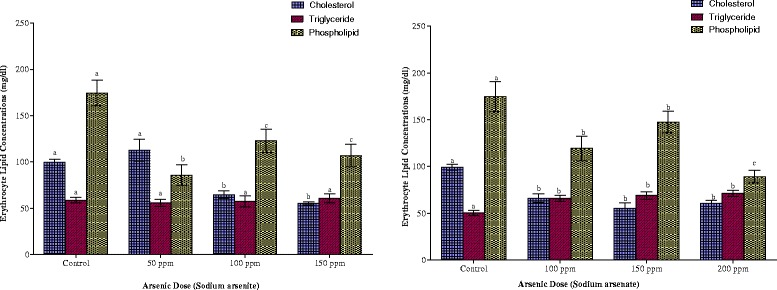
Effects of arsenite and arsenate on erythrocyte cholesterol, triglyceride and phospholipid concentrations. Each bar represents the mean ± S.E.M. of 7 rats. Bars with different alphabets are significantly different at p < 0.05

The erythrocyte lipid contents of the animals are shown in Fig. 5. Both arsenite and arsenate exposures resulted in significant depression of the cholesterol contents of the red blood cells in the animals (p < 0.05). Arsenite-exposure resulted in 35 % and 44 % decreases in cholesterol contents of the erythrocyte of rats with 100 ppm and 150 ppm As respectively. In arsenate-exposed rats, 34, 44 and 39 % reduction resulted from exposure to 100 ppm, 150 ppm and 200 ppm arsenic doses respectively. Arsenite exposure did not affect triglyceride contents of rat erythrocytes as no significant change (p > 0.05) was observed in the concentrations. Exposure to 100 ppm As in arsenate-exposed animals, however, resulted in a 31 % increase in erythrocyte triglyceride concentration (p < 0.05). Increase in the arsenic dose did not result in any further significant increase in the erythrocyte triglyceride.

Phospholipid concentrations in the erythrocyte were markedly lowered on exposure to the two arsenicals, although in both cases, the reduction was not dose-dependent. While the reduction amounted to 51, 29 and 39 % respectively in arsenite-treated animals, it amounted to 32, 16 and 49 % respectively in arsenate-treated animals.

**Fig. 6:**
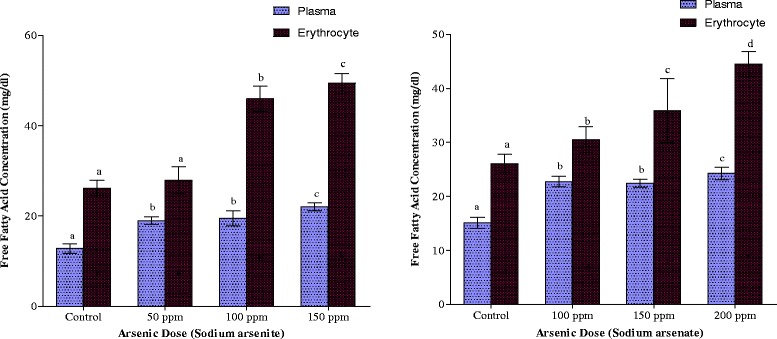
Effects of arsenite and arsenate on plasma and erythrocyte free fatty acid concentrations. Each bar represents the mean ± S.E.M. of 7 rats. Bars with different alphabets are significantly different at p < 0.05

Plasma and erythrocyte FFA concentrations are depicted in Fig. 6. In both arsenite- and arsenate-exposed animals, there was a gradual increase in both plasma and erythrocyte FFA concentrations as the arsenic doses were increased. Comparatively, in the plasma, the increase was more pronounced in arsenate-treated animals whereas in the erythrocytes the increase was more pronounced in arsenite-treated animals.

**Fig. 7:**
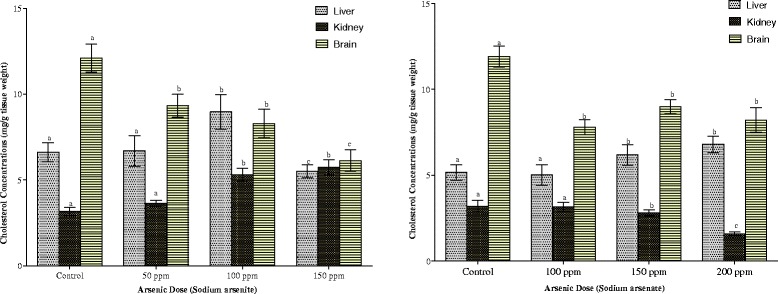
Effects of arsenite and arsenate on hepatic, renal and brain cholesterol concentrations. Each bar represents the mean ± S.E.M. of 7 rats. Bars with different alphabets are significantly different at p < 0.05

The effects of the arsenicals on hepatic, renal and brain cholesterol concentrations are depicted in Fig. 7. While arsenite increased hepatic and renal cholesterol by 35 and 82 % respectively, it decreased brain cholesterol by as much as 50 % at the highest dose of arsenic. Arsenate, on the other hand, increased hepatic cholesterol but decreased renal and brain cholesterol concentrations by 50 and 31 % respectively.

**Fig. 8:**
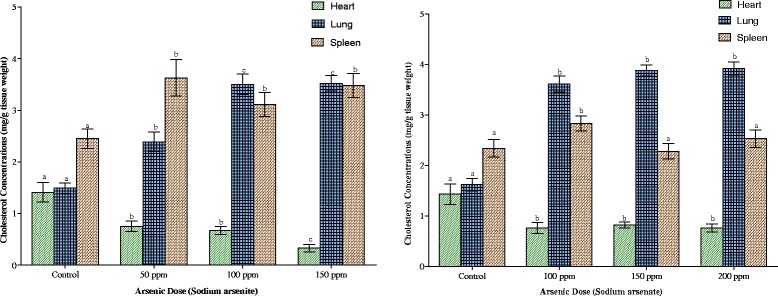
Effects of arsenite and arsenate on heart, lung and spleen cholesterol concentrations. Each bar represents the mean ± S.E.M. of 7 rats. Bars with different alphabets are significantly different at p < 0.05

Cardiac, pulmonary and splenic cholesterol contents are shown in Fig. 8. Exposure to the two arsenicals resulted in the depletion of the cardiac cholesterol while increasing pulmonary and splenic cholesterol contents. At the highest dose of each arsenical, cardiac cholesterol was just 23 % (arsenite) and 50 % (arsenate) of control values respectively. In the pulmonary compartment, the pattern of increase was similar for the two arsenicals. In the splenic compartment, however, while the highest increase (42 %) was observed with the highest dose of arsenic in arsenite-exposed animals, it was the 100 ppm As in arsenate-exposed animals that elicited the highest increase (21 %).

**Fig. 9:**
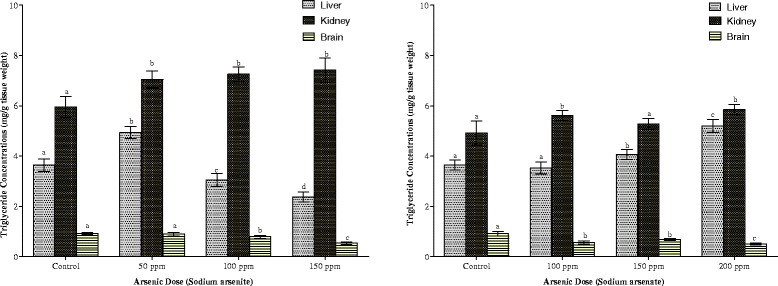
Effects of arsenite and arsenate on hepatic, renal and brain triglyceride concentrations. Each bar represents the mean ± S.E.M. of 7 rats. Bars with different alphabets are significantly different at p < 0.05

Arsenite elicited a hormetic response in hepatic triglyceride concentrations (Fig. 9). While the 50 ppm arsenic dose up-regulated triglyceride concentration by 35 %, the highest dose of arsenic (150 ppm) also caused a 35 % reduction in triglyceride concentration (Fig. 9). Arsenate, on the other hand, increased hepatic triglyceride (11 % and 43 %) at 150 and 200 ppm As doses respectively. Both arsenite and arsenate increased the renal contents of triglyceride. The increase was more pronounced in arsenite-treated animals. The two arsenicals also decreased to the same extent the triglyceride concentration of the brain, although the decrease was not dose-dependent.

**Fig. 10:**
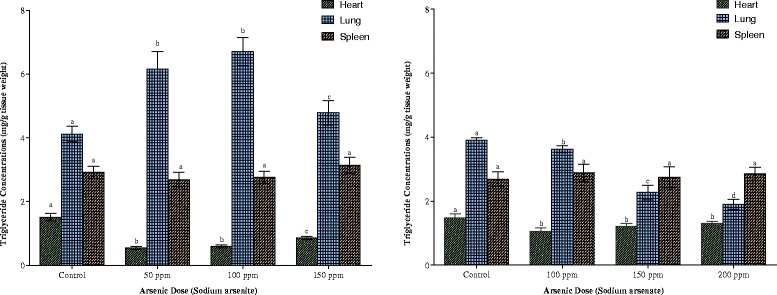
Effects of arsenite and arsenate on heart, lung and spleen triglyceride concentrations. Each bar represents the mean ± S.E.M. of 7 rats. Bars with different alphabets are significantly different at p < 0.05

Cardiac, pulmonary and splenic triglyceride contents are shown in Fig. 10. The heart responded to the presence of the two arsenicals in a similar pattern. While the heart responded to the lowest dose of each arsenical with a decrease (64 % in arsenite and 28 % in arsenate), triglyceride contents began to increase with each increasing dose of the arsenicals, although the increase did not reach statistical significance (p > 0.05) within the exposed groups. In contrast to the heart, there was a dose-dependent decrease in the triglyceride contents in the lungs in arsenate exposure. Although arsenite also increased pulmonary triglycerides, the increase was not dose-dependent. Exposure to the two arsenicals did not result in any significant change in splenic triglyceride.

**Fig. 11:**
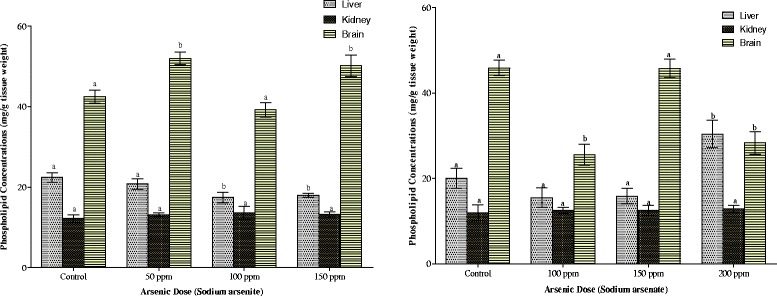
Effects of arsenite and arsenate on hepatic, renal and brain phospholipid concentrations. Each bar represents the mean ± S.E.M. of 7 rats. Bars with different alphabets are significantly different at p < 0.05

**Fig. 12:**
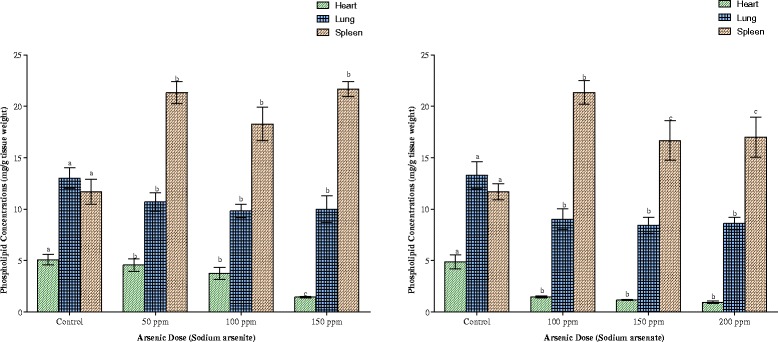
Effects of arsenite and arsenate on heart, lung and spleen phospholipid concentrations. Each bar represents the mean ± S.E.M. of 7 rats. Bars with different alphabets are significantly different at p < 0.05

The mean phospholipid concentrations in the tissues of the animals as depicted in Figs. 11 and 12 indicate that exposure to both arsenicals resulted in splenic phospholipidosis. Arsenite exposure alone resulted in brain phospholipidosis while arsenate exposure at the highest dose (200 ppm As) resulted in hepatic phospholipidosis. In contrast however, arsenate caused a significant reduction in phospholipid concentrations in the brain, heart and lung of the animals (Fig. 11) whereas it was only in the heart, liver and lung that phospholipid was reduced on exposure to arsenite (Figs. 11 and 12). Renal phospholipids were not affected by any of the arsenicals irrespective of the dose (Fig. 11).

**Fig. 13:**
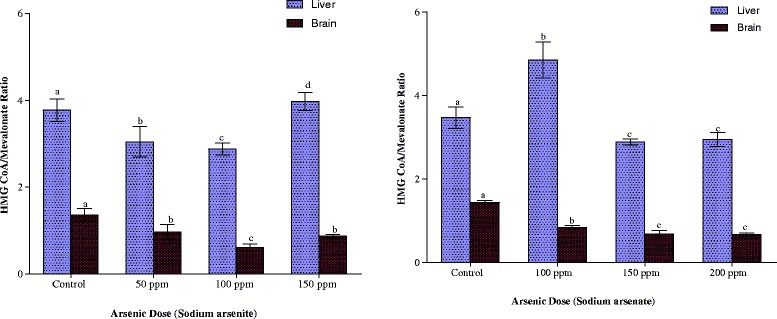
Effects of arsenite and arsenate on hepatic and brain HMG CoA/ Meva-lonate ratios as an Index of HMG CoA reductase activity. Each bar represents the mean ± S.E.M. of 7 rats. Bars with different alphabets are significantly different at p < 0.05

As indicated in Fig. 13, arsenite (150 ppm As) and arsenate (100 ppm As), inhibited hepatic HMG CoA reductase activity. At other doses of the arsenicals, hepatic HMG CoA reductase was up-regulated by 17 % by the two arsenicals respectively. The activity of the brain enzyme was also up-regulated by the two arsenicals (arsenite 56 % and arsenate 54 %).

The associations between tissue arsenic levels and some lipid parameters are depicted in Table 3. While tissue arsenic levels correlated positively with plasma FFA, reverse cholesterol transport was negatively correlated with tissue arsenic levels. Tissue arsenic levels (with the exception of hepatic arsenic vs. hepatic HMG CoA reductase and brain arsenic vs. hepatic HMG CoA reductase), also correlated positively with hepatic and brain HMG CoA reductase.

**Table 3:** Association between tissue arsenic levels and some lipid parameters in the animals on exposure to sodium arsenite and sodium arsenate for 12 weeks through drinking water

	Plasma FFA	HDL cholesterol	Hepatic HMG CoA reductase	Brain HMG CoA reductase
Parameter	Correlation coefficient (r)	Correlation coefficient (r)	Correlation coefficient (r)	Correlation coefficient (r)
Liver arsenic	0.491^a^	−0.634^a^	NS	−0.382^a^
Kidney arsenic	0.577^a^	−0.614^a^	−0.365^a^	−0.537^a^
Brain arsenic	0.602^a^	−0.611^a^	NS	−0.556^a^
Blood arsenic	0.578^a^	−0.732^a^	NS	−0.584^a^

^a^Significant at p < 0.05

NS-Not Significant

## Discussion

Arsenic accumulated in all the organs investigated irrespective of the chemical species administered with concentrations raised by many folds compared to control. The rather high level of arsenic in the blood of the exposed rats has been reported by others [13, 23] and is mainly contributed by the erythrocytes which are responsible for the distribution of arsenic throughout the body.

Following exposure to inorganic arsenic, As V is sequentially reduced to As III. The As III produced by this reduction or from direct ingestion is then oxidatively methylated to give pentavalent organic arsenicals, monomethylarsonic acid (MMA) and dimethylarsinic acid (DMA) [24]. This in turn is a function of cellular uptake of the inorganic arsenic in various tissues [25]. Uptake of substances in a cell is a function of the cell membranes. At physiological pH, trivalent arsenic compounds are neutral in charge while pentavalent arsenicals are negatively charged. Trivalent arsenic species can thus, readily transverse cell membranes than do pentavalent species [26]. Arsenite, therefore, might have been deposited in the organs at a faster rate than arsenate. Apart from blood, spleen accumulated the highest concentration of arsenic than any other tissue studied in arsenite-exposed rats. This was followed by heart and then kidney, brain, lung and liver in that order. In arsenate-exposed rats, accumulation was highest in the kidney, followed by lung, heart, brain, spleen and liver. Arsenate, in its protonated form, has similar properties and can substitute for phosphate in several biochemical reactions [27]. The high deposition in the kidney could, therefore, have resulted from the organ’s physiological involvement in the reabsorption of phosphate. Arsenate and phosphate have been reported to compete for the same binding sites, with a preference for arsenate absorption [28]. Once taken up by the tissues, inorganic arsenic is methylated to MMA^V^ and DMA^V^ and excreted in the urine [24]. However, this biomethylation process may become saturated due to the different levels of methyltransferases present in each tissue, resulting in a reduced rate of clearance and hence increased deposition in the tissues [29]. Furthermore, this biomethylation process (oxidative methylation followed by reduction to trivalency) which was originally considered a detoxification process, may actually be an activation process in view of the formation of reactive intermediates like MMA^III^ and DMA^III^ [30]. In addition to these, improvement in analytical techniques has led to the discovery of new sulphur-containing methylated arsenic metabolites, monomethylmonothioarsonic acid (MMMTA^V^) and dimethylmonothioarsinic acid (DMMTA^V^) in human urine [31]. To what extent this recent arsenic toxicokinetic information influences arsenic distribution/deposition in tissues on exposure to inorganic arsenic through drinking water remains to be elucidated.

The findings of this study also indicate that sub-chronic exposure to arsenite and arsenate through drinking water is associated with lipotoxic and non-lipotoxic perturbations in lipid homeostasis in organs, lipoproteins, plasma and erythrocytes as well as increase in HMG CoA reductase activity. Compared to control animals, lipotoxic perturbations in arsenic-exposed animals were characterised by high circulating FFA, enhanced splenic cholesterogenesis and phospholipidosis, pulmonary and hepatic cholesterogenesis as well as hypertriglyceridemia in plasma and LDL + VLDL fraction. Decrease in pulmonary and hepatic phospholipids, depletion of cardiac and brain lipids as well as depletion of erythrocyte, plasma and HDL cholesterol and phospholipids in circulation, characterised the non-lipotoxic effects of both arsenicals. An enrichment with cholesterol and depletion of phospholipids were also observed in the LDL + VLDL fraction. Since the patterns of this homeostatic imbalance differ with each arsenic specie and dose, different mechanisms might mediate this imbalance. In addition, since exposure to the arsenic species was through the oral route, interaction between arsenic and these lipids probably did not begin at the level of the gastro-intestinal tract. Rather, lipid dynamics after absorption might be the lipotoxic target of arsenic. These observations could be discussed along several lines.

Under normal physiological conditions, all tissues avidly acquire lipids from circulating non-esterified FFA associated with albumin, esterified fatty acids bound to lipoproteins chylomicrons and very low density lipoproteins (and liberated by lipoprotein lipase-mediated lipolysis), internalization of whole lipoproteins and *de novo* synthesis [32, 33], although *de novo* synthesis might play only a minor role in some tissues [32, 34]. Excess lipid, beyond that needed for cellular structures and ATP generation is stored in lipid droplets [32, 34]. However, tissues like liver, intestine, heart and brain are able to synthesise and secrete excess lipids in lipoproteins [32, 35–38].

FFAs originating from either albumin or lipoproteins enter the organs either by passive diffusion or via a protein carrier-mediated pathway [33, 34]. While the former is a lower affinity non-saturable process operating at higher FFA concentrations, the protein carrier-mediated pathway is a low capacity but high affinity process operating at FFA/albumin ratios found in the plasma [33, 34]. Protein carriers that have been characterized include fatty acid translocase (FAT/CD 36), plasma membrane fatty-acid binding protein and fatty acid transport protein [33, 34].

Both arsenic species induced significant elevation of plasma FFA concentration. This observation implies that while arsenic exposure did not affect the absorption of dietary lipids, cellular lipid dynamics was modulated by arsenic exposure. Generally hydrolysis of triglycerides occurs in the adipose tissue and results in the production of FFA with its subsequent release into the plasma. The elevation of this lipid suggests its increased mobilization from the adipose tissue which could be induced by physiological and psychological stress [39]. With consistent report of arsenic causing oxidative stress [40–42], the elevated plasma FFA implies an arsenic-induced augmentation of triglyceride hydrolysis in the adipose tissue by triglyceride lipase, resulting in increased mobilization of the liberated FFA into the plasma. The physiological consequences of this elevated plasma FFA could be viewed from the metabolic roles of FFA. While this elevated plasma FFA should provide an immediate substrate for triglyceride synthesis as well as the source of available fuel for the tissues and also the necessary signal for tissues to oxidize them [43, 44], data in Fig. 6 indicate what while exposure to arsenic did not inhibit the uptake of FFA by the tissues, a considerable amount of the FFA was directed towards the synthesis of triglycerides in the liver, kidney and lungs as well as phospholipids in the spleen. Data in Fig. 9 also indicate that while the liver could be said to have a limited capacity for triglyceride storage, tissues like kidney and lungs accumulated the triglyceride to about 2-fold that of the liver. This further suggests that arsenic induced a dysfunction of triglyceride degradation secondary to insufficient mitochondrial ß-oxidation of FFA, thus compromising energy metabolism in these tissues. Further significant energetic and functional consequences might be expected.

The key enzyme in the distribution of circulating lipids between organs is lipoprotein lipase (LPL), an enzyme located on the walls of blood capillaries [32]. The role of LPL in lipoprotein metabolism is well known. Since majority of the circulating FFAs are present as triglycerides in lipoproteins, hydrolysis of this triglyceride by LPL is an important determinant of overall fatty acid uptake and ß-oxidation in the tissues [45, 46]. High circulating FFA (as observed in this study) is known to inhibit the activity of LPL [47]. Homeostatically, inhibition of LPL is expected to have the following consequences on lipid dynamics in the circulation:

1. Severe hypertriglyceridemia secondary to underutilisation of chylomicrons and VLDL
2. Hypocholesterolemia
3. Decrease in HDL cholesterol (otherwise known as reverse cholesterol transport) and phospholipids [48].

That arsenic exposure resulted in all these lipid metabolic derangements (Figs. 1 and 3) suggests that the inhibition of LPL might mediate these observed effects of arsenic.

Another major finding of this study was the depletion of cardiac lipids in arsenic-exposed animals. Physiologically, cardiac myocytes have regulatory pathways that regulate lipid metabolism. The myocardium has labile stores of triglyceride that serve as an endogenous source of FFAs [49, 50]. Intramyocardial triglyceride can be hydrolysed by hormone sensitive lipase and adipose triglyceride lipase [32, 33]. Insulin inhibits lipolysis, whereas catecholamines, thyroid hormone and glucagon, accelerate intramyocardial triglyceride degradation [51]. Whether arsenic-induced adrenergic stress in the animals might mediate the depletion of cardiac lipids observed in this study is not known at present. Furthermore, since the major cardiac lipids were depleted as a result of arsenic exposure, the possibility that arsenic might induce overexpression/production of cardiac apolipoprotein B (Apo B) which increased lipid secretion from the heart, cannot be ruled out [38]. Apo B expression has been found to increase in hearts of patients with coronary artery disease [52], a cardiovascular outcome that is a hallmark of arsenic exposure [53].

Due to the blood brain barrier, circulating lipoproteins cannot reach the brain except for small HDL particles [54]. Most of the lipoproteins in the brain are synthesised by the astrocytes and have been postulated to be responsible for the transfer of lipids within the brain [36, 55]. Thus, an arsenic-induced damage to the blood brain barrier might be responsible for the dyslipidemia observed in the brain of the arsenic-exposed animals [56, 57].

Enhanced cholesterogenesis observed in the spleen, lungs and liver of arsenite- and arsenate-exposed animals as well as the kidney of arsenate-exposed animals, may be attributed to an arsenic-induced activation of 3-hydroxy-3-methylglutaryl Coenzyme A (HMG CoA) reductase (the rate limiting enzyme in cholesterol synthesis) or it may be due to feedback inhibition [58, 59]. It may also be due to inhibition of the activity of cholesterol-7α-hydroxylase, a cytochrome P450 enzyme located in the endoplasmic reticulum. This could limit the biosynthesis of bile acids, which is the only significant route for elimination of cholesterol from the body [60]. Since the liver has limited capacity to store lipids, the excess cholesterol and triglycerides are packaged into VLDL particles and secreted into circulation. Consistent with this was the observation of the enrichment of LDL + VLDL fraction with cholesterol and triglycerides. Furthermore, since this fraction was depleted of phospholipids, this implies a failure occasioned by arsenic exposure in hepatic provision of phospholipids that are found in lipoproteins (Fig. 4) [61].

Compelling experimental data from animal studies have shown a positive association between high circulating FFA and pathological conditions such as diabetes mellitus, obesity, thyrotoxicosis, atherosclerosis, coronary artery disease, cardiac arrhythmia, high blood pressure and pulmonary diseases [39, 62–64]. Furthermore, elevated pulmonary cholesterol has been shown to inhibit surfactant function by disrupting the physiology and turnover of surfactant, leading to impairment of lungs mechanics [65]. Abnormalities in surfactant metabolism are the leading causes of acute respiratory distress syndrome, acute lung injury and a diverse array of other respiratory illnesses [66]. That arsenic exposure causes all these conditions have been confirmed from epidemiological studies of arsenicosis [3]. Thus, the sustained dyslipidemia observed in this study may be one of the underlying mechanisms of these arsenic-induced pathologies.

One limitation of this study was that arsenic speciation was not carried out in the tissues. Rather, arsenic in the tissues was determined as total arsenic. In view of the recent discovery of new methylsulphur derivatives of inorganic arsenic, it opens up the possibility that these organic metabolites may be more subtle and insidious in their toxic effects. Toxicological information on these newly discovered organic metabolites is scanty. However, in a recent study in our laboratory in which rats were exposed to pentavalent inorganic and organic arsenicals through drinking water, arsenic accumulation and dyslipidemia of the same magnitude were also observed. These recent data, together with the data in the present study, support a linkage between metabolism and chemical form of arsenic and lipotoxic and non-lipotoxic effects of this metalloid.

## Conclusion

In conclusion, even though sub-chronic exposure to arsenite and arsenate through drinking water produced different patterns of dyslipidemia, our study identified two common denominators of dyslipidemia namely: inhibition of reverse cholesterol transport and increase in plasma FFA. These two denominators (in addition to other individual perturbations of lipid metabolism induced by each arsenical), might mediate the observed cardiovascular and other disease endpoints of inorganic arsenic exposure through drinking water.

## Abbreviations

HDL: High density lipoproteins
FFAs: Free fatty acids
HMG CoA reductase: 3-hydroxy-3-methylglutaryl coenzyme A reductase
LDL: Low density lipoproteins
VLDL: Very low density lipoproteins
MMA: Monomethylarsonic acid
DMA: Dimethylarsinic acid
MMMTA: Monomethylmonothioarsonic acid
DMMTA: Dimethylmonothioarsinic acid
FAT: Fatty acid translocase
LPL: Lipoprotein lipase
